# Treatment and Chest Reconstruction for Mediastinitis Following Sternotomy for Cardiac Surgery at the Heart Institute of the University of São Paulo Medical School

**DOI:** 10.21470/1678-9741-2020-0117

**Published:** 2021

**Authors:** Vitor Penteado Figueiredo Pagotto, Samuel Terra Gallafrio, Igor Castro Carneiro, Rolf Gemperli, Fabio B. Jatene

**Affiliations:** 1 Plastic Surgery Division, Universidade de São Paulo, São Paulo, São Paulo, Brazil.; 2 Cardiothoracic Surgery Department, Universidade de São Paulo, São Paulo, São Paulo, Brazil.

**Keywords:** Mediastinitis, Negative-Pressure Wound Therapy, Debridement, Sternum, Surgical Flaps, Cardiac Surgical Procedures, Infection Control.

## Abstract

This study presents the method used for chest reconstruction and treatment of mediastinitis following cardiac surgery at the Heart Institute of the University of São Paulo Medical School. After infection control with antibiotic therapy associated with aggressive surgical debridement and negative pressure wound therapy, chest reconstruction is performed using flaps. The advantages and disadvantages of negative pressure wound therapy are discussed, as well as options for flap-based chest reconstruction according to the characteristics of the patient and sternum. Further studies are needed to provide evidence to support the decisions when facing this great challenge.

**Table:** 

Abbreviations, acronyms & symbols	
ICC	= Infection Control Committee
Incor-FMUSP	= Heart Institute of the University of São Paulo Medical School
LD	= Latissimus dorsi
NPWT	= Negative pressure wound therapy
VRAM	= Vertical rectus abdominis myocutaneous

## INTRODUCTION

Median sternotomy has been established as the gold standard access to cardiac surgery. However, surgical site infection and the development of mediastinitis remain two of the main complications related to the procedure. The incidence of mediastinitis varies between 1 to 5% in cardiac surgeries and this severe infection is related to increased morbidity, mortality, and length of hospital stay, with a consequent increase in the overall cost of treatment^[^^1^^]^.

The continuing advancement of medical knowledge has enabled the development of new options of treatment over the last few years, improving drastically the treatment of mediastinitis, which has ceased to be a feared complication to become a problem that can be controlled. The advent of negative pressure wound therapy (NPWT) in the 1990s as an adjunctive method to treat this complication was a major contributor to this paradigm shift^[^^1^^]^.

The Heart Institute of the University of São Paulo Medical School (Incor-FMUSP) performs about 3,000 cardiac procedures and 190,000 outpatient consultations annually. As presented by other publications, the incidence of mediastinitis after median sternotomy was 1.3% in 2012^[^^2^^]^. As a result of this high number of cases, the treatment of this severe infection represents a great challenge at the institute. In this study, we present the treatment and final chest reconstruction for mediastinitis following sternotomy for cardiac surgery at Incor-FMUSP.

## TECHNIQUE

The mediastinitis's treatment is initiated as soon as the diagnosis is performed, based on clinical, laboratory, and/or imaging tests, according to the guidelines of the Centers for Disease Control and Prevention (Table 1)^[^^3^^]^. Empirical antibiotic therapy guided by the Infection Control Committee (ICC) is the first measure.

**Table 1:** Mediastinitis criteria^[^^3^^]^.

Mediastinitis must meet at least one of the following criteria:	
1	Patient has organism(s) identified from mediastinal tissue or fluid by a culture or non-culture based microbiologic testing method which is performed for purposes of clinical diagnosis or treatment, for example, not Active Surveillance Culture/Testing (or ASC/AST).
2	Patient has evidence of mediastinitis on gross anatomic or histopathologic exam.
3	Patient has at least one of the following signs or symptoms: fever (> 38.0°C), chest pain*, or sternal instability*. And at least one of the following: a. Purulent drainage from mediastinal area. b. Mediastinal widening on imaging test.
4	Patient ≤ 1 year of age has at least one of the following signs or symptoms: fever (> 38.0°C), hypothermia (< 36.0°C), apnea*, bradycardia*, or sternal instability*. And at least one of the following: a. Purulent drainage from mediastinal area. b. Mediastinal widening on imaging test.

* With no other recognized cause

Comment: The mediastinal space is the area under the sternum and in front of the vertebral column, containing the heart and its large vessels, trachea, esophagus, thymus, lymph nodes, and other structures and tissues. It is divided into anterior, middle, posterior, and superior regions

Surgical treatment consists of aggressive debridement of all infected and devitalized tissue in the first procedure, including soft and bone tissue. Samples are then collected for culture and antibiograms. When the sternum presents instability or laxity of the steel wires, samples of bone fragments are also collected for culture and pathology for diagnosis of osteomyelitis. Thereafter, NPWT at 125 mmHg is installed continuously.

After five to seven days, the patient returns to the operating room for wound revision, with copious lavage and debridement of any residual suspicious tissue. Again, continuous NPWT at 125 mmHg is performed.

**Fig. 1:**
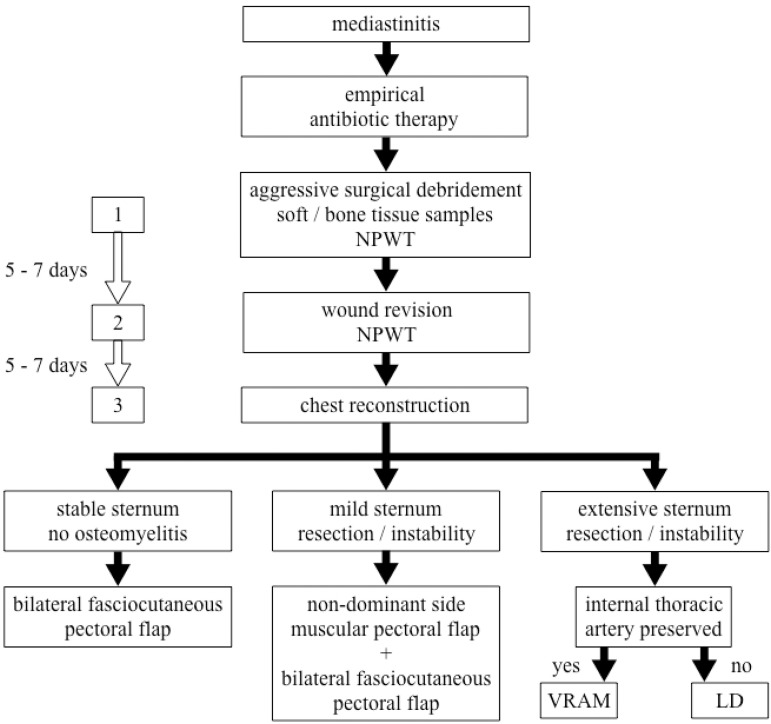
The flowchart elucidates the choice of the method of chest reconstruction according to the conditions of the sternum, the presence of osteomyelitis, and the preservation of the internal thoracic artery. LD=latissimus dorsi flap; NPWT=negative pressure wound therapy; VRAM=vertical rectus abdominis myocutaneous flap

Finally, after another interval of five to seven days, the patient returns to the operating room for the third time. At this point, significant clinical and laboratory improvement is expected, as well as culture-guided antibiotics and enhancement of the wound aspect. Steel wires are retained if they still have any effect on sternum stabilization. The reconstruction of the wound is then performed according to the sternum’s conditions (Figure 1).

**Fig. 2:**
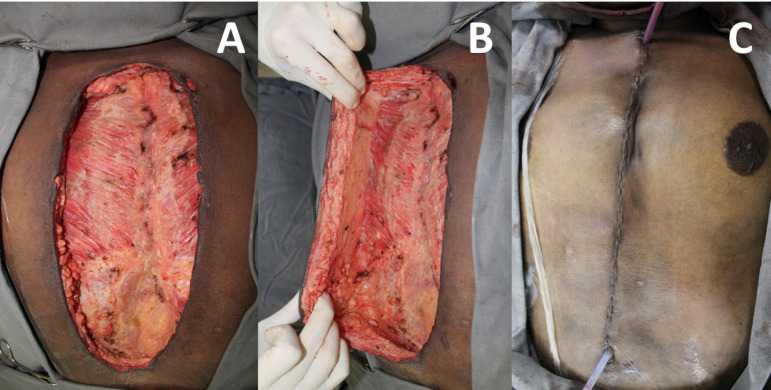
Patient with mediastinitis, stable sternum, and no associated osteomyelitis. A) After antibiotic therapy, surgical debridement, lavage, and negative pressure therapy, the wound presents reconstruction conditions. B) Dissection of a bilateral pectoral fasciocutaneous flap. The skin, subcutaneous tissue, and pectoral fascia are separated from muscle tissue along the entire length of the wound, allowing the flaps to advance over the sternal wound. C) The final aspect of the reconstruction is shown.

Patients with stable sternum and no associated osteomyelitis are reconstructed with the advancement of bilateral pectoral fasciocutaneous flap. This alternative ensures adequate wound coverage with well-vascularized and viable tissue without the need for new surgical access and with less risk of adverse effects of muscle transfer, such as hematoma and decreased upper limb strength. It is also a fast and less complex procedure^[^^4^^]^ (Figure 2).

**Fig. 3:**
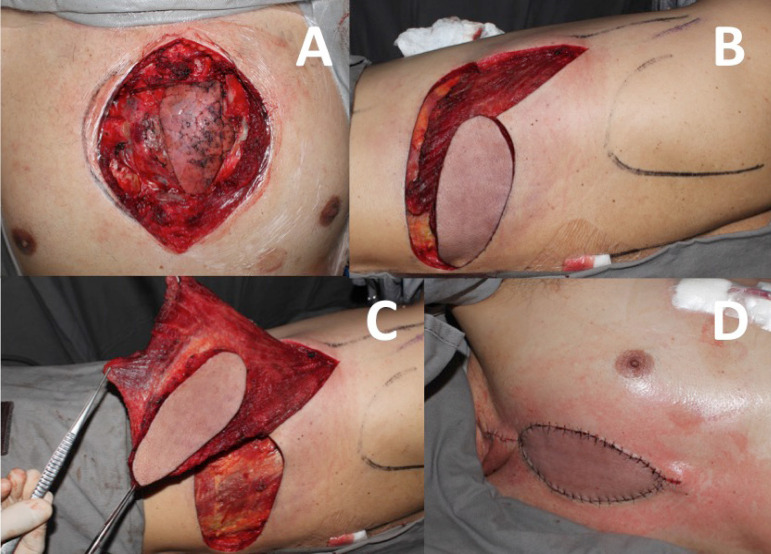
A) Patient with total sternum dehiscence and associated mediastinitis after myocardial revascularization surgery. B) After aggressive surgical debridement, the sternum is viable and is fixed with steel wires. C) After antibiotic therapy and negative pressure therapy, the sternum persists with mild instability in the lower and upper portions, but the wound presents reconstruction conditions. D) The pectoralis major muscle is dissected from the non-dominant side with the release of its lower margin. E) The muscle is also detached from the rib cage and clavicle, with the preservation of the pectoral branch of the thoracoacromial artery (arrow). F) The flap is then advanced over the sternum, allowing adequate coverage of the wound. G) Final aspect of the reconstruction without additional scarring.

When the patient remains with some degree of sternal instability or bone loss after debridement, reconstruction is performed with a unilateral pectoralis major muscle flap on the non-dominant side, associated with the advancement of the bilateral pectoral fasciocutaneous flap (Figure 3).

**Fig. 4:**
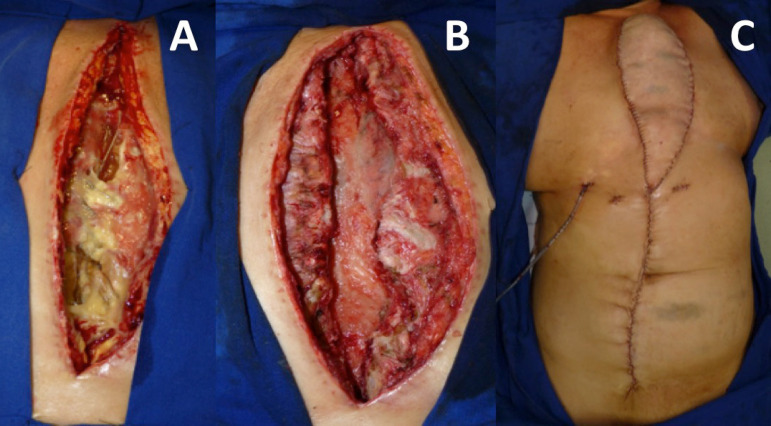
A) Patient has sternum dehiscence and mediastinitis, with impaired bone tissue viability. B) After antibiotic therapy, aggressive surgical debridement, including total resection of the sternum, lavage, and negative pressure therapy, the wound presents reconstruction conditions. C) A vertical rectus abdominis myocutaneous (VRAM) flap was performed to provide a large amount of tissue to protect the mediastinum. It is not necessary to mobilize the patient because of the surgery.

**Fig. 5:**
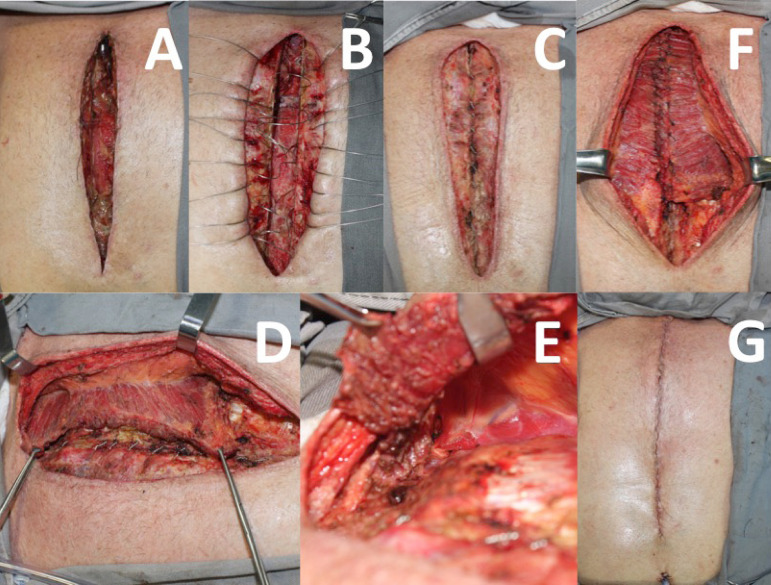
A) Resulting wound after complete resection of the sternum. B) The patient is positioned in the right lateral decubitus to make a vertical incision in the posterior axillary line and a horizontal incision in the lower edge of the skin island for dissection of the left latissimus dorsi flap. C) After complete dissection of the muscle flap with preservation of the skin island, the flap can be mobilized to the anterior chest through a subcutaneous tunnel. D) Final aspect of the reconstruction: chest reconstruction with large amounts of tissue to protect the mediastinum.

In cases of extensive sternal osteomyelitis requiring total or subtotal resection, wound reconstruction is preferably performed with a rectus abdominis myocutaneous flap with vertical skin island (or vertical rectus abdominis myocutaneous [VRAM] flap) (Figure 4). In situations where bilateral internal thoracic arteries are not available, for example, because of their use as artery bypass grafting, the latissimus dorsi myocutaneous flap can be used. Sternal resection exposes mediastinal structures more sharply, and therefore, flaps that allow more tissue transfer are preferred^[^^4^^]^ (Figure 5).

## DISCUSSION

Several risk factors are related to the development of mediastinitis. The main patient risk factors are age, obesity, smoking, diabetes, chronic kidney disease, chronic obstructive pulmonary disease, and malnutrition. The risk factors resulting from the surgical procedure are the use of internal thoracic artery graft, duration of surgery and cardiopulmonary bypass, use of bone wax, emergency surgery, and reoperation. Finally, poor glycemic control and prolonged postoperative orotracheal intubation are also related to the increased risk of mediastinitis^[^^4^^,^^5^^]^.

The main microorganisms responsible for mediastinitis are *Staphylococcus epidermidis* and *Staphylococcus aureus*, arising from contamination of the surgical site or the patient's microbiota, as well as *Klebsiella* and *enterobacteria*, arising from translocation of the respiratory, genitourinary, and gastrointestinal tract^[^^5^^]^.

Once the diagnosis of mediastinitis is performed, empirical antibiotic therapy should be started with coverage for Gram-positive, Gram-negative, and anaerobic bacteria. Nevertheless, antibiotic therapy guided by culture should be the standard^[^^5^^]^. At Incor-FMUSP, the initial empirical treatment recommended by the ICC is usually performed by vancomycin in combination with piperacillin and tazobactam.

Surgical treatment consists of extensive surgical debridement followed by NPWT until the reconstruction of the wound is achieved. Treatment should be individualized based on the microorganism, the patient's clinical condition, and the extent of the injury^[^^4^^,^^6^^]^.

NPWT has emerged as a fundamental method in the treatment of mediastinitis. Applying subatmospheric pressure to the wound bed through a system of a plastic film-sealed polyurethane sponge connected to a pump ensures a sterile and stable chest environment, allowing early extubation, patient mobilization, and improved respiratory and hemodynamic functions^[^^1^^,^^4^^,^^5^^]^. The macro and microscopic deformations caused by this system allow drainage of wound secretion, reduction of edema, and reduction of the number of microorganisms. Besides that, it increases blood flow, promotes approximation between wound edges, stimulates granulation, and accelerates healing^[^^1^^,^^5^^]^.

Although there is no evidence to support the choice of pressure levels for the treatment of mediastinitis, 125 or 150 mmHg are able to promote the positive effects of NPWT and are the main values recommended by expert consensus^[^^7^^]^. The most feared risk of using this device is erosion and hemorrhage when in direct contact with the heart and large vessels. This situation can be overcome by placing a barrier between these vital structures and the sponge^[^^1^^]^. Despite its high cost, there are currently no cost-effectiveness studies for the treatment of mediastinitis with NPWT.

The use of NPWT after initial broad debridement allows for a shorter surgical time by avoiding immediate reconstruction of the wound and decreases surgical trauma in often critical and septic patients, allowing the return to the intensive care unit for improvement of clinical status. Thus, patients undergoing this treatment as a bridge for reconstruction have shorter hospital stays, shorter time for wound reconstruction, and, especially, lower mortality^[^^1^^,^^5^^]^.

Most patients with mediastinitis have a considerable loss of soft tissue and/or sternum, requiring flaps for chest reconstruction. Tissue transfer aims to protect the vital structures of the mediastinum, functional rehabilitation with sternum stabilization, and dead-space filling, in addition to seeking a satisfactory aesthetic result. The main flaps used for this purpose are the pectoralis major, rectus abdominis, and latissimus dorsi^[^^4^^,^^6^^]^.

The pectoralis major flap is the workhorse for chest reconstruction after mediastinitis. Its privileged location concerning the wound and its safe blood supply, as well as constant anatomical aspects, based on the thoracoacromial artery and perforating branches of the intercostal and internal thoracic arteries, when present, allow the procedure to be performed without new access incision, with good aesthetic and reproducible results. It can be performed bilaterally or unilaterally, preferably composed of myocutaneous tissues, due to the major surgical trauma of mobilizing only muscle tissue. Its primary disadvantage is that it provides insufficient coverage of the distal portion of the sternum and the xiphoid appendix. However, the use of NPWT favors coverage of this region without the need for an additional procedure. The main complications related to the pectoral flap are seroma, hematoma, chronic pain, and decreased upper limb strength, which is more frequent when muscle flaps with humeral disinsertion are performed. The fasciocutaneous flap promotes adequate wound coverage without the need for new surgical access and the adverse effects of muscle transfer^[^^4^^,^^6^^,^^8^^]^. The standard procedure at Incor-FMUSP is the unilateral flap on the non-dominant side without humeral disinsertion.

The rectus abdominis flap can be performed with transverse (the transverse rectus abdominis myocutaneous, or TRAM) or vertical (VRAM) skin island. Although technically reproducible, it requires an extensive incision in the donor site and can cause weakening of the abdominal wall with the development of hernias. The main indications are unavailability of the pectoralis major, insufficient coverage of the distal portion of the sternum, resection of the sternum due to severe osteomyelitis, or recurrence of infection. Importantly, the vascular supply of this flap is guaranteed by the superior epigastric artery, a continuous branch of the internal thoracic artery, which is often used for cardiac revascularization, especially the left one. Although the use of this flap based on intercostal or muscle-phrenic branches is described, this alternative is still used with restriction^[^^4^^,^^9^^]^.

Finally, a latissimus dorsi flap can be used in muscle or myocutaneous form. It allows the transfer of large amounts of muscle tissue to cover extensive defects without interfering with sternal and parasternal vascularization. However, it requires mobilization of the patient position and extensive incision in the donor site^[^^4^^,^^10^^]^.

## CONCLUSION

The treatment of mediastinitis after sternotomy for cardiac surgery is still a major challenge. Despite the advances provided by flap-based reconstruction and the incorporation of NPWT, the multiple presentations of this infection associated with the unique characteristics of each patient require individualized choices. Further studies are needed to provide evidence and elucidate the choice of the best methods of treatment and reconstruction.

**Table:** 

Authors' roles & responsibilities	
VPFP	Substantial contributions to the conception or design of the work; or the acquisition, analysis, or interpretation of data for the work; final approval of the version to be published
STG	Substantial contributions to the conception or design of the work; drafting the work or revising it critically for important intellectual content; agreement to be accountable for all aspects of the work in ensuring that issues related to the accuracy and integrity of any part of the work are appropriately investigated and resolved; final approval of the version to be published
ICC	Substantial contributions to the analysis or interpretation of data for the work; final approval of the version to be published
RG	Final approval of the version to be published
FBJ	Final approval of the version to be published
